# Supplementary material for: Low phosphate activates STOP1-ALMT1 to rapidly inhibit root cell elongation
Source: Nat Commun. 2017 May 15;8:15300. doi: 10.1038/ncomms15300 (PMC5440667; doi:10.1038/ncomms15300)
Supplement: Supplementary Information — Supplementary Figures and Supplementary Table [file ncomms15300-s1.pdf]

# Supplementary Information

**a**

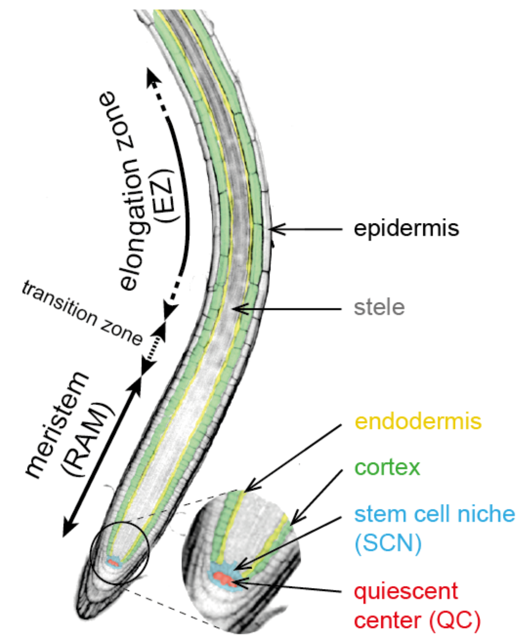


**b**

| Lab number | Genomic position from ATG | prediction |
|---|---|---|
| *almt1* alleles | | |
| *almt1*$^{1}$ | G974A | Defective splicing of intron 3 (3') |
| *almt1*$^{32}$ | G1201A | W255stop |
| *almt1*$^{45}$ | G334A | G71E |
| *almt1*$^{51}$ | G881A | Defective splicing of intron 3 (5') |
| *almt1*$^{61}$ | C471T | Q117stop |
| *almt1*$^{69}$ | G3A | M1I (translational frameshift at next ATG |
| *almt1*$^{140}$ | G782A | C176Y |
| *almt1*$^{154}$ | G816A | W187stop |
| *stop1* alleles | | |
| *Stop1*$^{25}$ | C1207T | Q403stop |
| *Stop1*$^{48}$ | C1297T | Q433stop |
| *stop1*$^{60}$ | G36A | W12stop |
| *stop1*$^{67}$ | G36A | W12stop |
| *stop1*$^{90}$ | C322A | Q108stop |
| *stop1*$^{115}$ | G122A | W41stop |
| *stop1*$^{136}$ | C784T | H262Y |
| *stop1*$^{139}$ | G1057A | E353K |
| *stop1*$^{141}$ | C934T | Q312stop |
| *stop1*$^{148}$ | G793A | G265R |

**c**

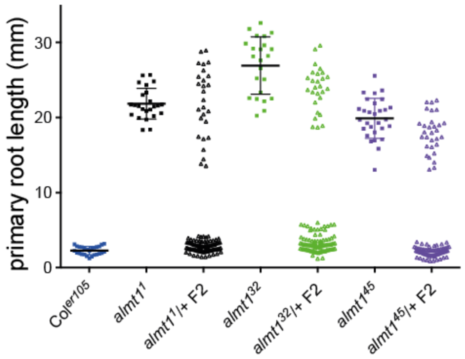


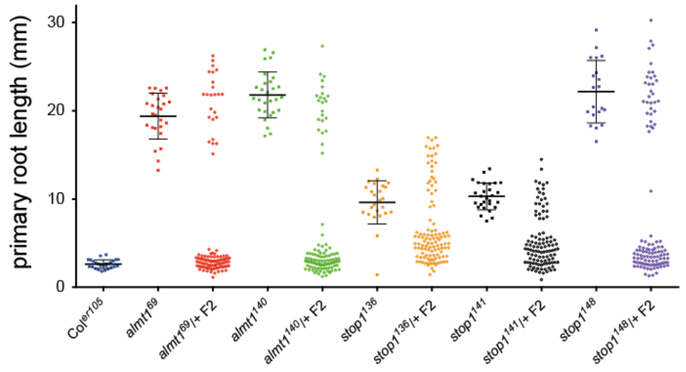


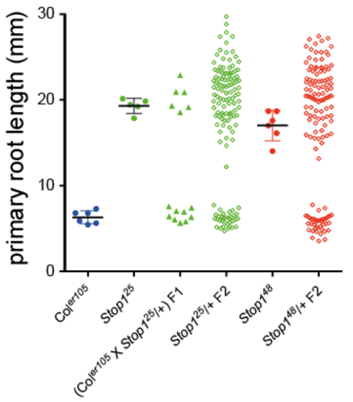


**d**

| populations | observed segregation | | | expected segregation (WT:mutant) | *P* (binomial test) | Different from expected segregation? (alpha<0.05) |
|---|---|---|---|---|---|---|
| | [mutant] | [WT] | total | | | |
| *almt1*$^{1}$/+ F2 | 28 | 94 | 122 | 3:1 | 0,3433 | no |
| *almt1*$^{32}$/+ F2 | 27 | 93 | 120 | 3:1 | 0,3036 | no |
| *almt1*$^{45}$/+ F2 | 31 | 87 | 118 | 3:1 | 0,4093 | no |
| *almt1*$^{69}$/+ F2 | 24 | 89 | 113 | 3:1 | 0,2094 | no |
| *almt1*$^{140}$/+ F2 | 23 | 91 | 114 | 3:1 | 0,1389 | no |
| | | | | | | |
| (♀Col$^{er105}$ X ♂*stop1*$^{25}$/+) F1 | 6 | 9 | 15 | 1:1 | 0,3036 | no |
| *stop1*$^{25}$/+ F2 | 105 | 37 | 142 | 1:3 | 0,4171 | no |
| *stop1*$^{48}$/+ F2 | 101 | 40 | 141 | 1:3 | 0,2028 | no |
| *stop1*$^{136}$/+ F2 | 27 | 91 | 118 | 3:1 | 0,3408 | no |
| *stop1*$^{141}$/+ F2 | 26 | 90 | 116 | 3:1 | 0,3005 | no |
| *stop1*$^{148}$/+ F2 | 34 | 83 | 117 | 3:1 | 0,1812 | no |

**Supplementary Figure 1: Genetic analysis of *almt1* and *stop1* mutations.**

(a) Scheme depicting the different cell types of the primary root tip.

(b-d) Genetic analysis of the *almt1* and *stop1* mutations.

(b) Table of the molecular lesions of *almt1* and *stop1* alleles.

(c, d) Segregation pattern of the mutant phenotypes in F1 or F2 populations from a cross between mutant and WT (+). Seedlings were grown for 7 days under –Pi prior to measuring the primary root length (mean (not for segregating populations) +/- s.d., *n* = 5–29 seedlings per line) (b), and statistical analysis of segregation patterns (c).

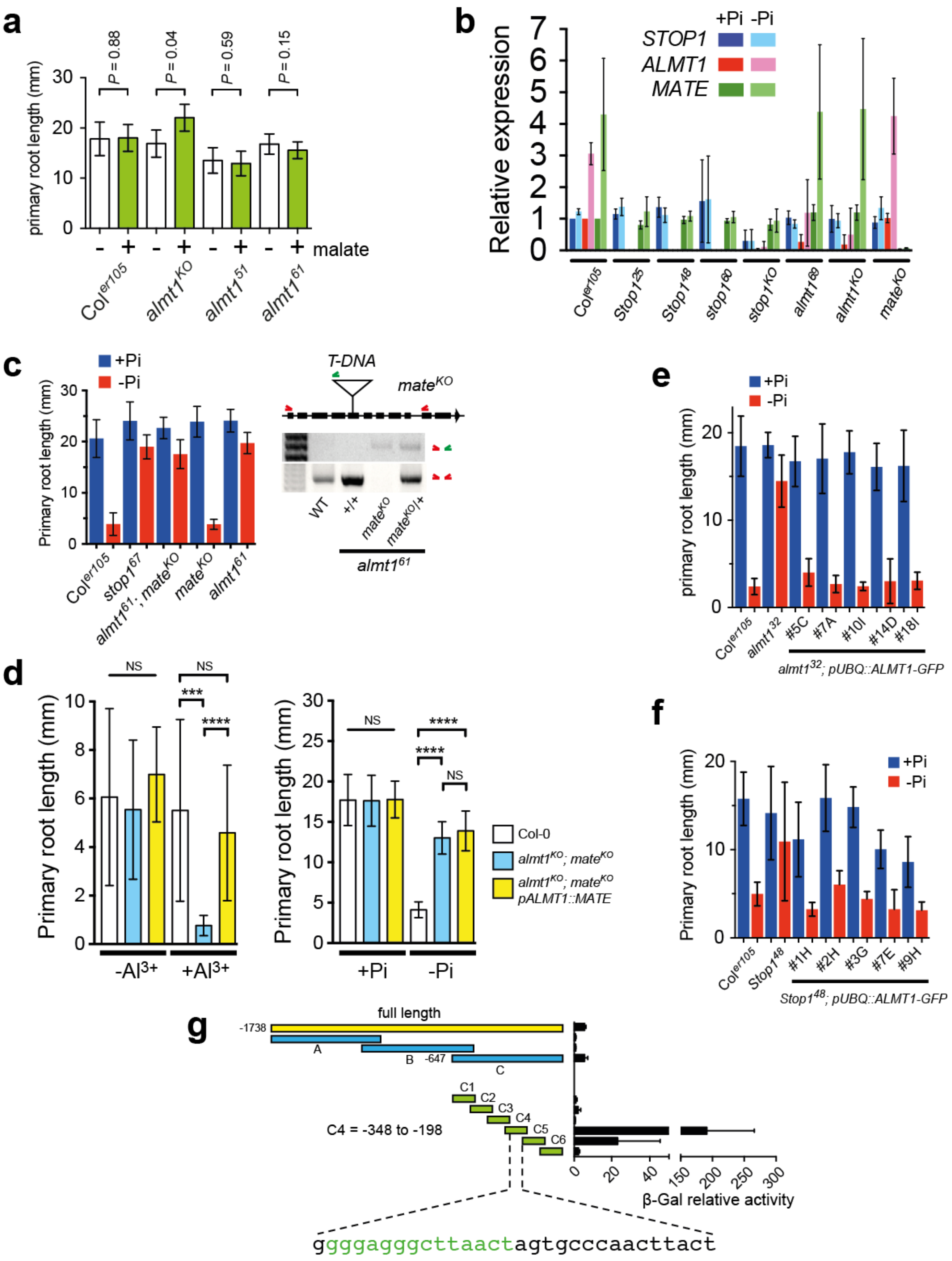


**Supplementary Figure 2: Expression of *STOP1*, *ALMT1* and *MATE*, and complementation analysis.**

(a) Under +Pi, exogenous malate does not inhibit primary root growth. WT and *almt1* seedlings grown under +Pi for 2 days were transferred for 6 days to +Pi with or without 200 μM malate (mean +/- s.d., *n* = 7–10 seedlings per condition).

(b) Gene expression analysis. Seedlings of the indicated genotypes were grown for 5 days under +Pi and transferred for 24 h to +Pi or –Pi medium and roots collected for RNA extraction followed by qRT-PCR on *STOP1*, *ALMT1* and *MATE* genes (mean +/- s.d., *n* = 3 independent experiments, each with three technical replicates). Expression levels are normalized relatively to the WT ($Col^{er105}$) control grown under +Pi.

(c) Analysis of the $mate^{KO}$ lines under –Pi.
Top: Primary root length of the $mate^{KO}$ and $almt1^{61}$;$mate^{KO}$ mutants. Seedlings were grown for 5 days under +Pi or –Pi (mean +/- s.d., *n* = 8–18 seedlings per line and condition). The experiment was performed twice with consistent results; one experiment is shown. Bottom: genotyping of the $mate^{KO}$ mutant. PCR results with the indicated primers (red and green arrowheads; see Supplementary Tab. 1 for sequences) on genomic DNA from the WT ($Col^{er105}$) and from $almt1^{61}$ lines with the indicated genotypes at the *MATE* locus (+/+, WT; $mate^{KO}$, homozygous KO mutant; $mate^{KO/+}$, heterozygous).

(d) *pALMT1::MATE* rescues the $almt1^{KO}$;$mate^{KO}$ double mutant for resistance to $Al^{3+}$ but not for the response to –Pi. Left: seedlings were grown five days in 0 or 4 μM $Al^{3+}$ (*n* = 10-18). Right: seedlings were grown six days in + or –Pi (*n* = 33-39). Mean +/- s.d.; two-tailed *t*-test, **** P < 0.0001; *** P < 0.001; NS, not significant. The experiment was performed twice with consistent results; one experiment is shown.

(e) Complementation of $almt1^{32}$ mutant with the *pUBQ::ALMT1-GFP* construct (five independent transgenic lines). Seedlings were grown for 5 days under –Pi and the primary root lengths measured (mean +/- s.d., *n* = 11–16 seedlings per line and condition). The experiment was performed twice with consistent results; one experiment is shown.

(f) Complementation of $Stop1^{48}$ mutant with the *pUBQ::ALMT1-GFP* construct

(five independent transgenic lines). Seedlings were grown for 5 days under –Pi and the primary root lengths measured (mean +/- s.d., *n* = 4–8 seedlings per line and condition).

(g) Yeast one-hybrid assay of STOP1 and *pALMT1*. Coloured bars represent parts of *pALMT1* fused to LacZ used in the assay; they are numbered with respect to first ATG of the *ALMT1* coding sequence. β-Gal activity is calculated relatively to the density of cells (see Methods) (mean +/- s.d., *n* = 3 independent experiments, each with two biological replicates per condition). The sequence in green indicates the STOP1-binding site found previously[28].

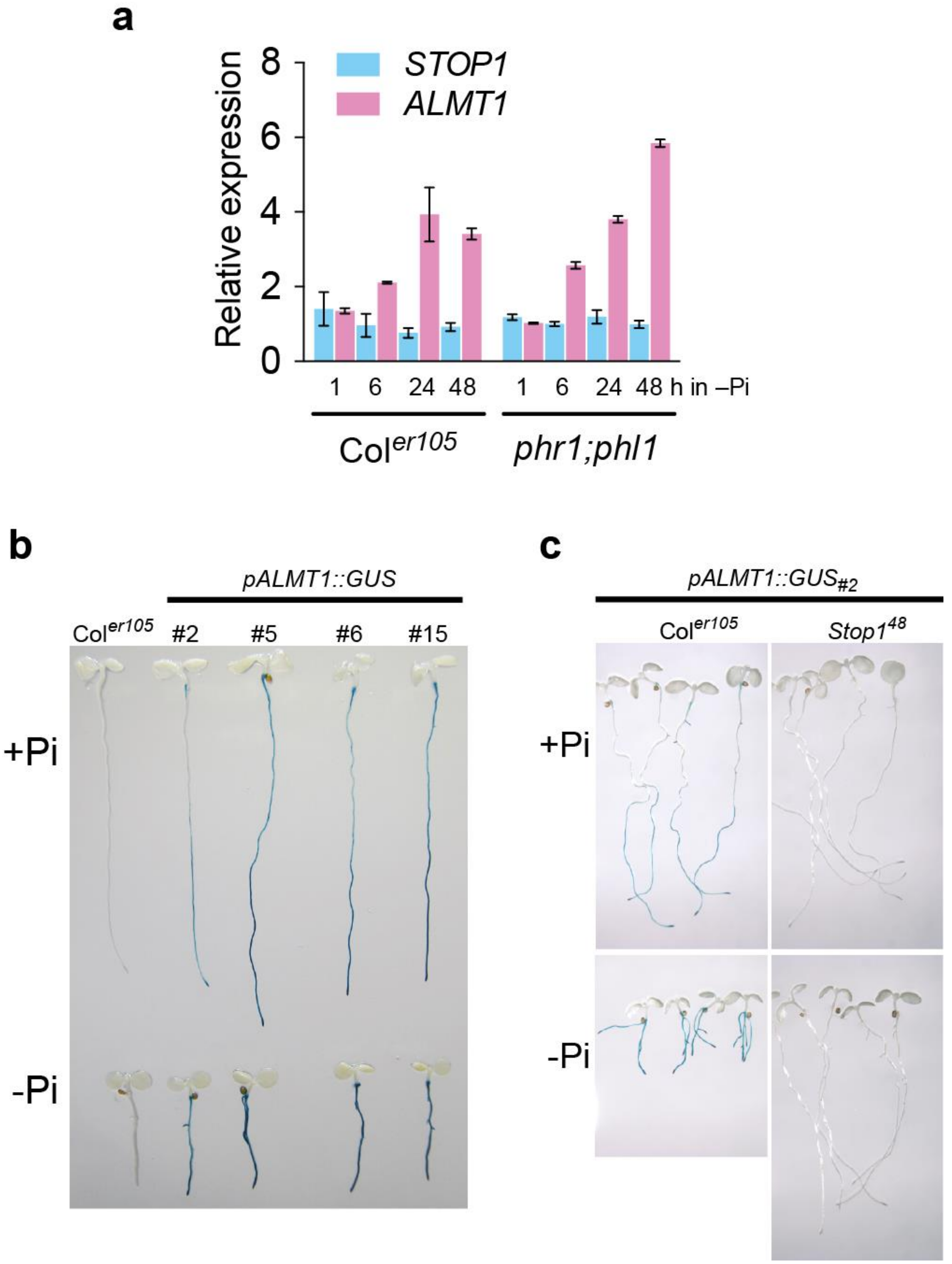


**Supplementary Figure 3: *ALMT1* expression in different genetics backgrounds.**

(a) Kinetics of *STOP1* and *ALMT1* expression (RT-qPCR) in WT and the *phr1;phl1* double mutant. Seedlings grown 5 days under +Pi were transferred for 1 to 48 h to –Pi. (mean +/- s.d.).

(b,c) Pattern of *ALMT1* expression in the primary root.
(b) Representative seedlings of four independent lines homozygous for the *pALMT1::GUS* construct were grown for 6 days under +Pi or –Pi prior the GUS staining (1h). Untransformed WT ($Col^{er105}$) is shown as a negative control of the GUS staining. Two independent experiments were performed with consistent results and one representative experiment is shown.

(c) $Stop1^{48}$ abolishes the expression of *ALMT1*. WT ($Col^{er105}$) and $Stop1^{48}$ seedlings homozygous for the $pALMT1::GUS_{\#2}$ construct were grown for 6 days under +Pi or –Pi prior the GUS staining. Two independent experiments were performed with consistent results and one representative experiment is shown.

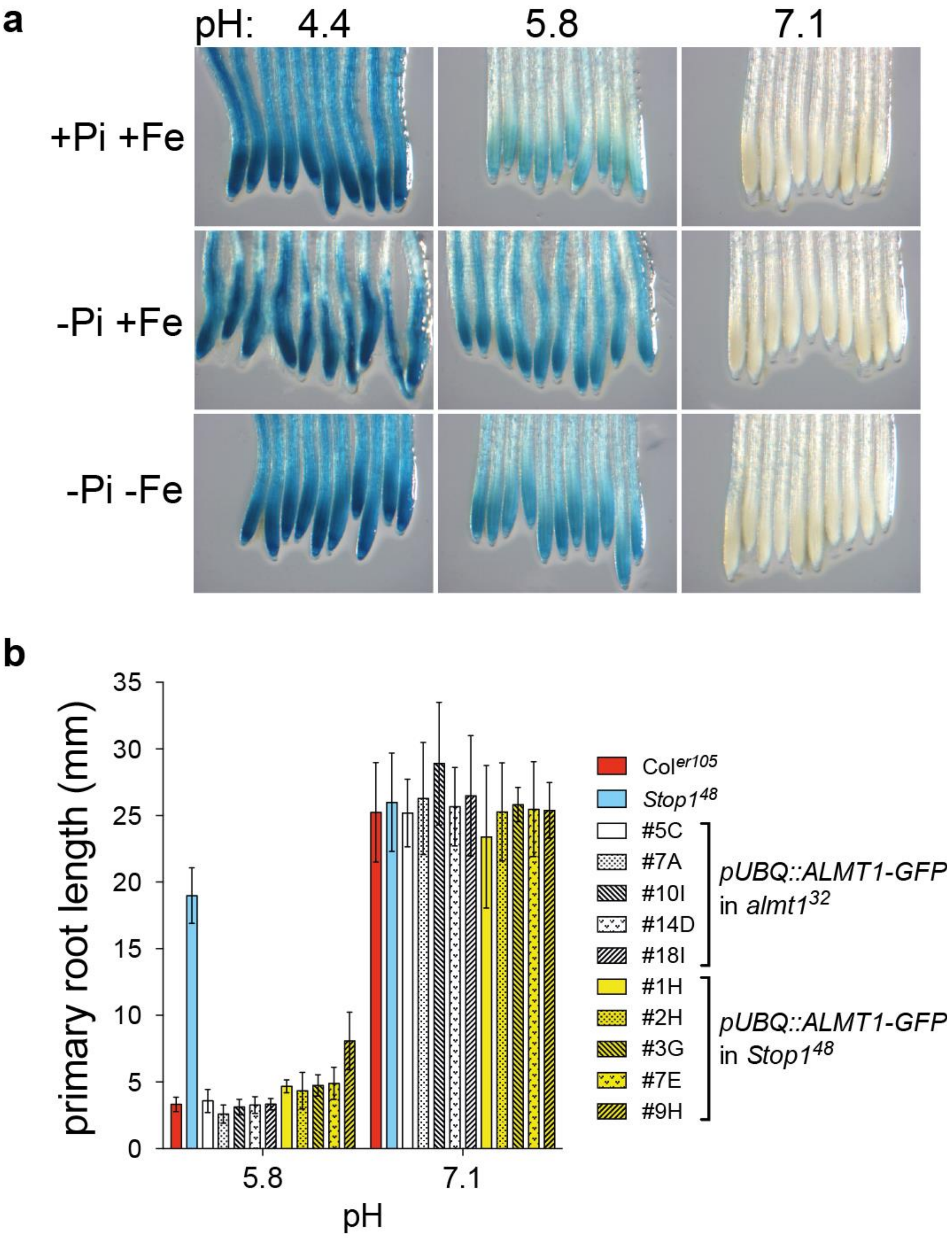


**Supplementary Figure 4: Effect of the pH on the expression of *ALMT1* and on the primary root growth under –Pi.**

(a) *pALMT1::GUS* expression at different pH.

Four-day old *pALMT1::GUS*$_{\#2}$ seedlings grown under +Pi at pH 7.1 were transferred 48 h in + or –Pi at the indicated pH before GUS staining. Two independent experiments were performed with consistent results and one representative experiment is shown.

(b) At pH 7.1, the constitutive expression of *ALMT1* does not promote a short root under –Pi. The *UBQ::ALMT1-GFP* construct has been introduced in the *almt1*$^{32}$ and *Stop1*$^{48}$ mutants and five independent transgenic lines selected. Homozygous seedlings were sown on –Pi+Fe plates at pH 5.8 or 7.1 and the primary root lengths measured after 6 days (mean +/- s.d., *n* = 8-15). Note that at pH 5.8, the *UBQ::ALMT1-GFP* construct complements both the *almt1*$^{32}$ and *Stop1*$^{48}$ mutants, but it does not confer a short primary root at pH 7.1.

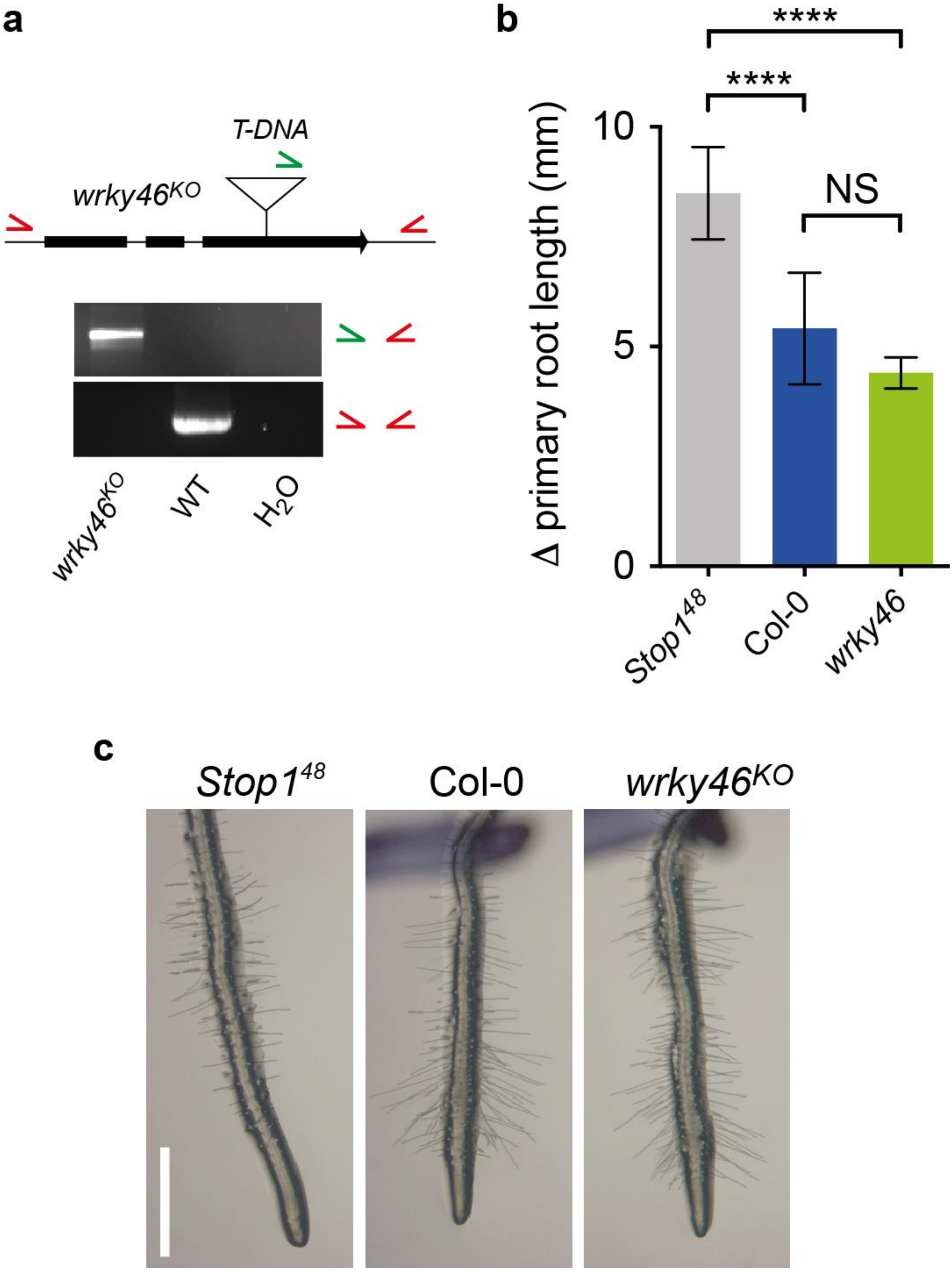


**Supplementary Figure 5: Analysis of the *wrky46*$^{KO}$ mutant under –Pi.**

(a) Genotyping of the *wrky46*$^{KO}$ mutant. PCR results with the indicated primers (red and green arrowheads; see Supplementary Tab. 1 for sequences) on genomic DNA from the WT (Col-0) and the *wrky46*$^{KO}$ mutant.

(b) Δ primary root length of the *Stop1*$^{48}$, WT (Col-0) and *wrky46*$^{KO}$ lines. Four-day old seedlings grown under +Pi were transferred 48 h under –Pi before measuring root length and photographing the root tip (mean +/-s.d., n = 5-10; two-tailed *t*-test; **** $P<0.0001$; NS, not significant).

(c) Pictures of the root tip of the indicated lines grown in (b). Scale, 1mm.

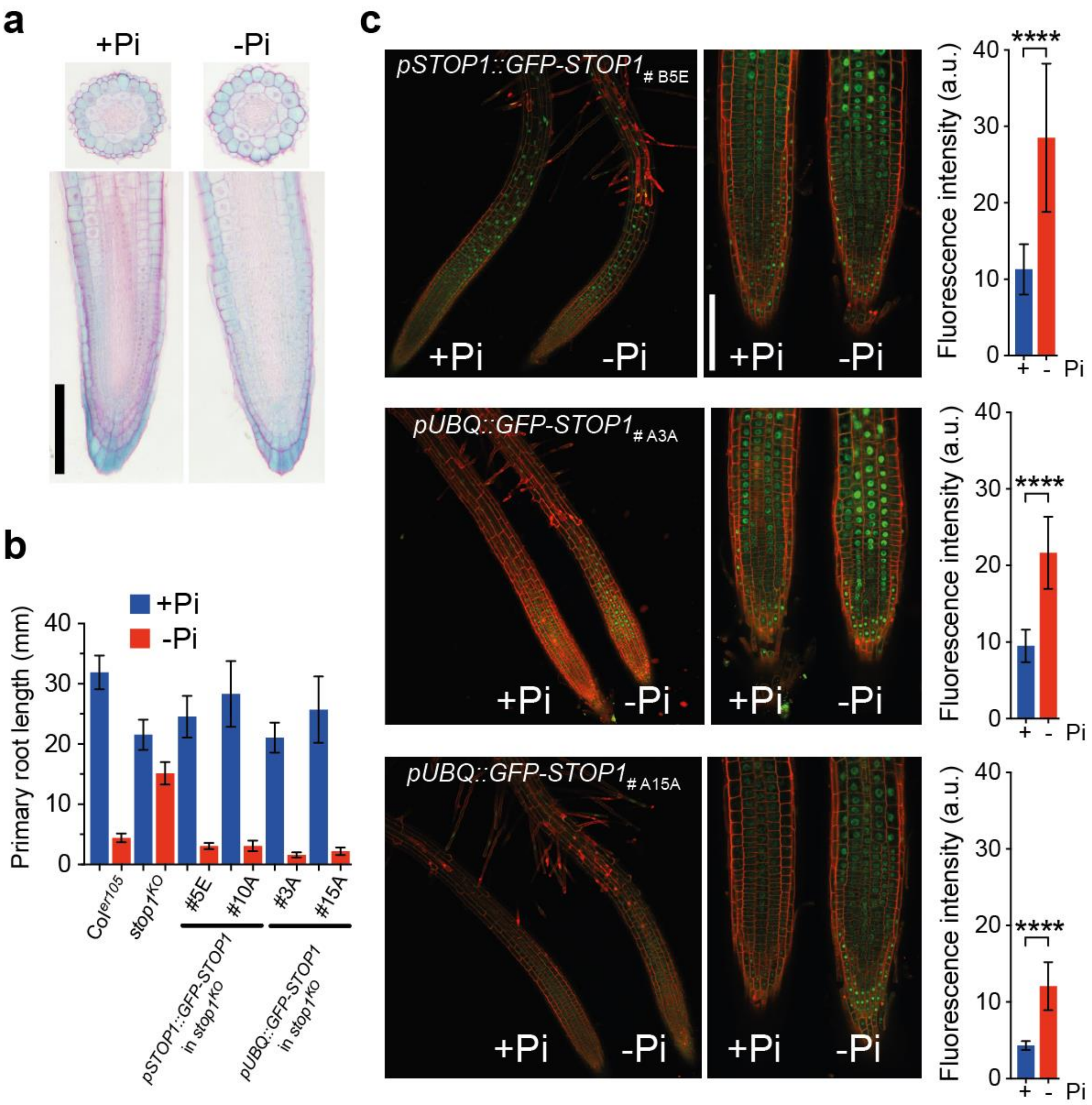


**Supplementary Figure 6: Expression of STOP1.**

(a) Pattern of *STOP1* expression in the primary root tip. Transverse (top) and longitudinal (bottom) sections of the root tip of *pSTOP1::GUS* (in WT). Seedlings were grown for 5 days under +Pi and transferred for 48 h to +Pi or –Pi prior GUS staining. Two independent experiments were performed with consistent results and one representative experiment is shown. Scale bar, 100 μm.

(b) Complementation of the *stop1*$^{KO}$ mutant with *pSTOP1::GFP-STOP1* or *pUBQ::GFP-STOP1* constructs (2 independent transgenic lines per construct). Seedlings were grown for 6 days to +Pi or –Pi medium and the primary root lengths measured (mean +/- s.d., *n* = 10–15 seedlings per line and condition).

(c) GFP-fluorescence of GFP-STOP1 in the primary root tip. Five day-old *stop1*$^{KO}$;*pSTOP1::GFP-STOP1* (1 line, independent from that in Fig. 1g) and *stop1*$^{KO}$;*pUBQ::GFP-STOP1* (2 independent lines) seedlings grown under +Pi were transferred for 24 h +Pi or –Pi plates and GFP-fluorescence pictured with confocal microscope. All pictures were taken with same microscope and camera settings as in Fig.1g. Left pictures: the two roots were mounted side by side on the same microscope slide. Middle pictures: magnification of the root tips. Three independent experiments were performed with consistent results and one representative experiment is shown. Right panels: intensity of the GFP-fluorescence (a.u., arbitrary units) in nuclei of the root tip (mean +/- s.d., $n$ = 29-60 nuclei; two-tailed $t$-test; **** $P$<0.0001). Scale bar, 100 μm.

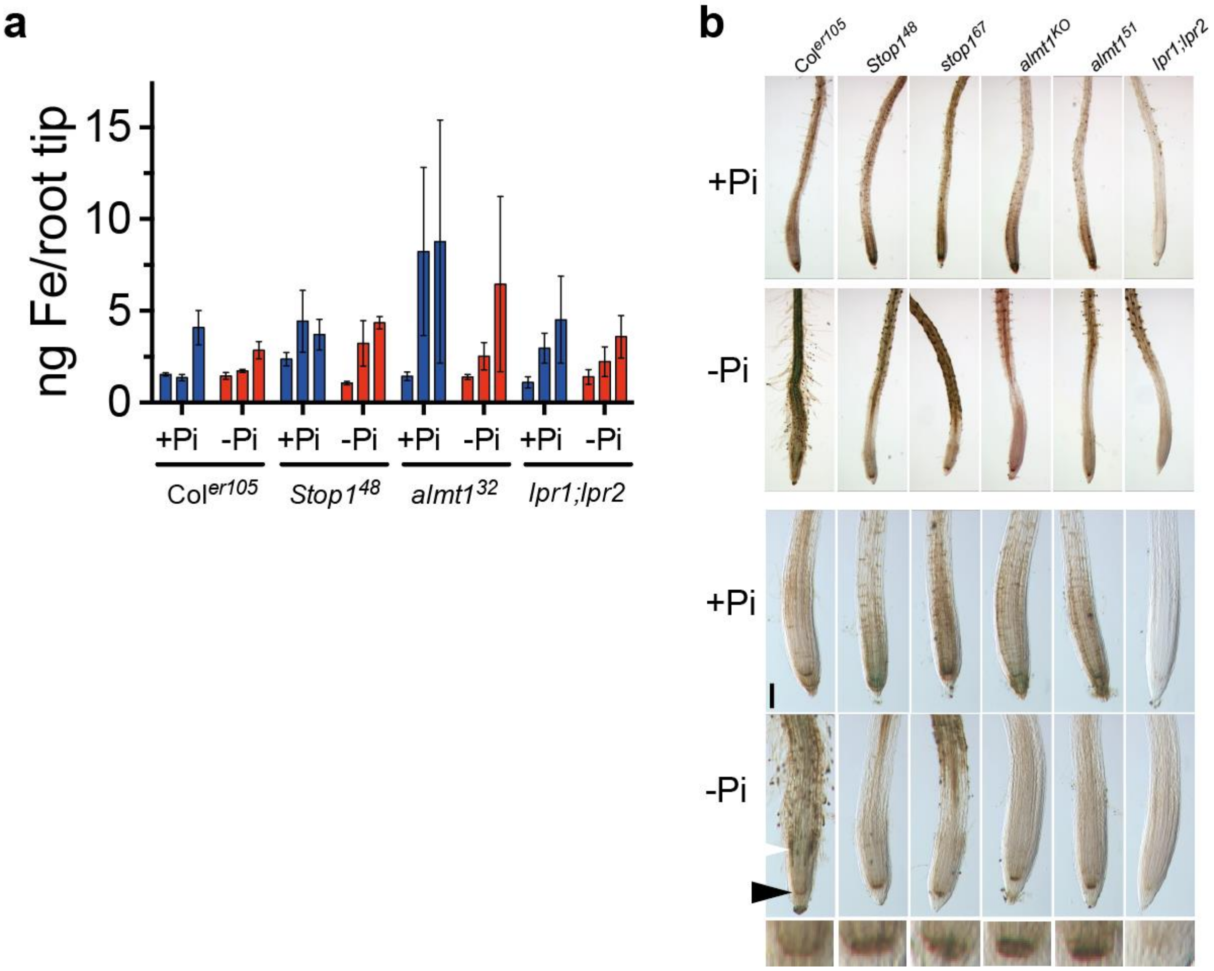


**Supplementary Figure 7: Fe localisation in root tips.**

(a) Iron content in the primary root tip. Seedlings of the indicated genotypes were grown for 5 days under +Pi and transferred for 24 h to +Pi or –Pi prior to harvesting root tips (≈5 mm) for ICP-MS measurements of iron (mean +/-s.e.m., *n* = 3 independent experiments, each with 3 biological replicates of 60 root tips each).

(b) Histochemical detection of Fe accumulation and distribution in the primary root of WT and mutants (top panel) and the apex, with magnification of the SCN under –Pi (bottom panels). Three-day old seedlings of the indicated genotypes were transferred 48 h in + or –Pi plates prior to Perls/DAB staining. Note the accumulation of Fe in the elongation zone of the WT (white arrowhead) and in the SCN (black arrowhead). Three independent experiments were performed with consistent results and one representative experiment is shown. Scale bar, 100 μm.

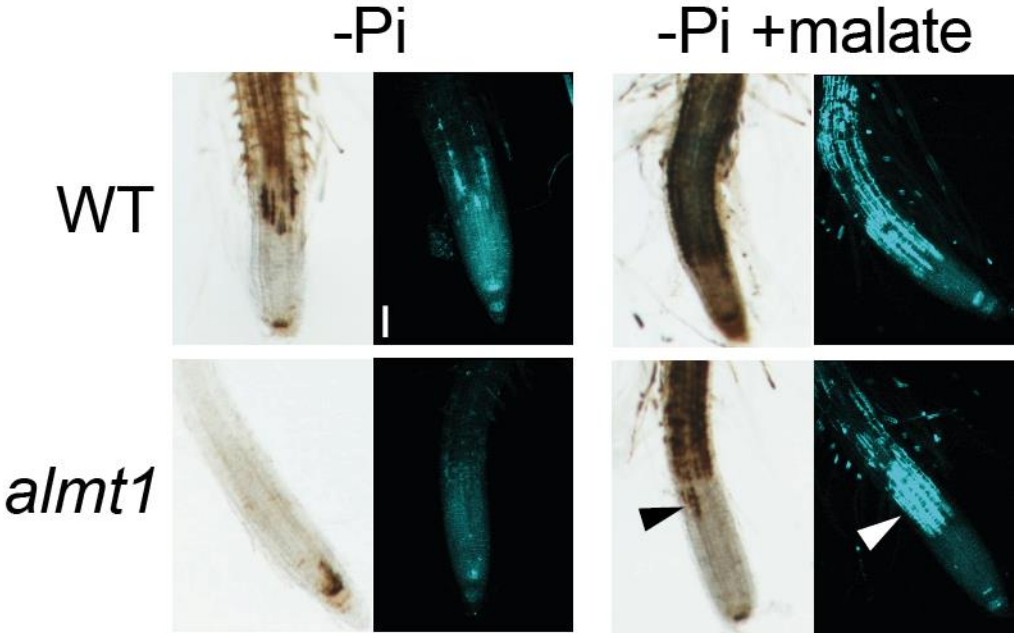


**Supplementary Figure 8: Malate complementation of the *almt1* mutant.**

Four-day old WT and *almt1* seedlings grown under +Pi were transferred 1 day under -Pi or -Pi supplemented with 250 µM malate before staining iron (left panels) or callose (right panels). Note the accumulation of iron (black arrowhead) and callose (white arrowhead) in the elongation zone of the *almt1* grown under –Pi+malate. Scale bar, 100 µm.

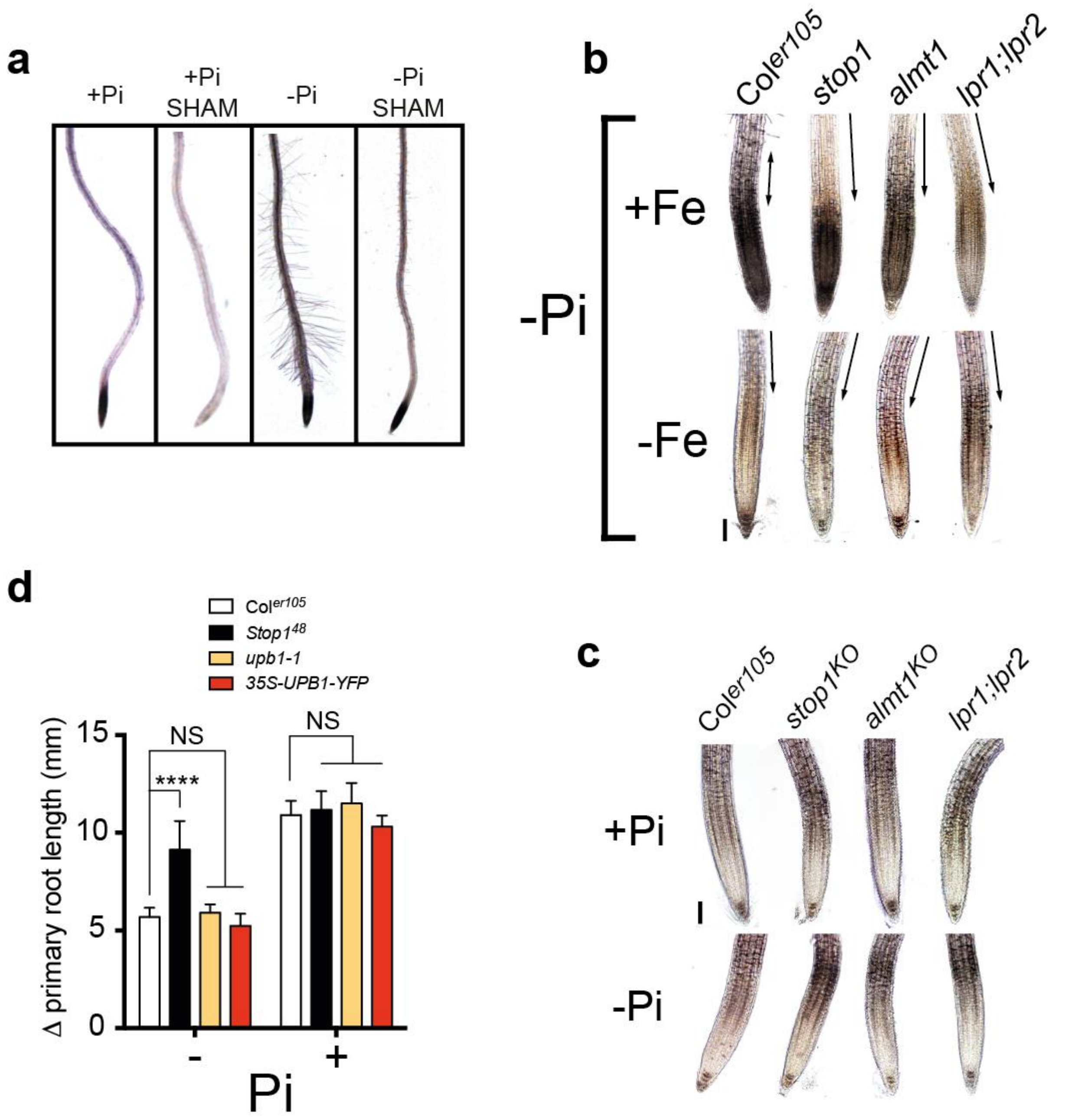


**Supplementary Figure 9: Peroxidase activity in the primary root of mutants.**

(a) Histochemical detection of peroxidase activity in the WT primary root. Four-day-old WT seedlings were transferred for 48 h to +Pi or –Pi plates, with or without 15 μM SHAM prior to staining with 4-chloro-1-naphtol.

(b) Effect of iron on the peroxidase activity in the primary root tip. Seedlings of the indicated genotypes grown for 6 days under +Pi were transferred for 24 h to –Pi, +Pi or -10 μM Fe, prior staining with 4-chloro-1-naphtol. Arrows indicate the position of

the elongation zone. The experiment was performed three times with consistent results; one representative experiment is shown. Scale bar, 100 μm.

(c) Effect of neutral pH on the peroxidase activity in the primary root tip. Four-day-old seedlings of the indicated genotypes were transferred for 48 h to +Pi or –Pi, pH 7.1 plates prior staining with 4-chloro-1-naphtol. The same lines grown at pH 5.8 are shown in Fig. 4k. The experiment was performed three times with consistent results; one representative experiment is shown. Scale bar, 100 μm.

(d) Primary root length of *upb1-1* and *35S-UPB1-YFP* lines. Three-day-old seedlings were transferred for 2 days to +Pi or –Pi, (mean +/- s.d., *n* = 8–10 seedlings per line and condition; two-way ANOVA, multiple comparisons; **** $P$<0.0001).

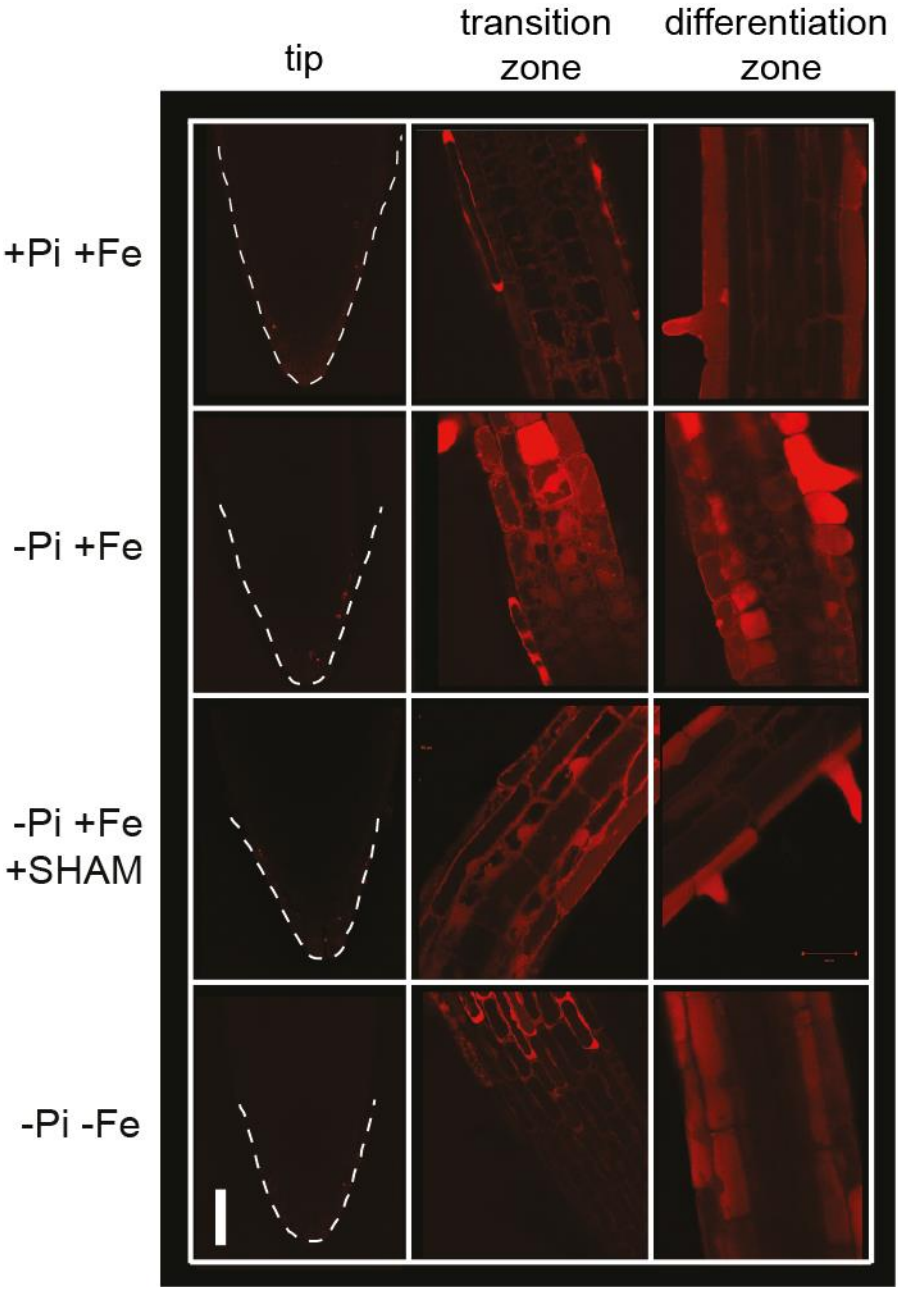


**Supplementary Figure 10: ROS detection in the root tip.**

Six-day old Col$^{er105}$ seedlings grown under +Pi were transferred for 24 h in the indicated conditions before staining ROS with CM-H2DCFDA. SHAM was at 15 μM. Pictures were taken in the root tip (left), in the transition zone (middle) and in the differentiation zone (right). The dashed white lines indicate position of the root tips. Scale bar, 50 μm.

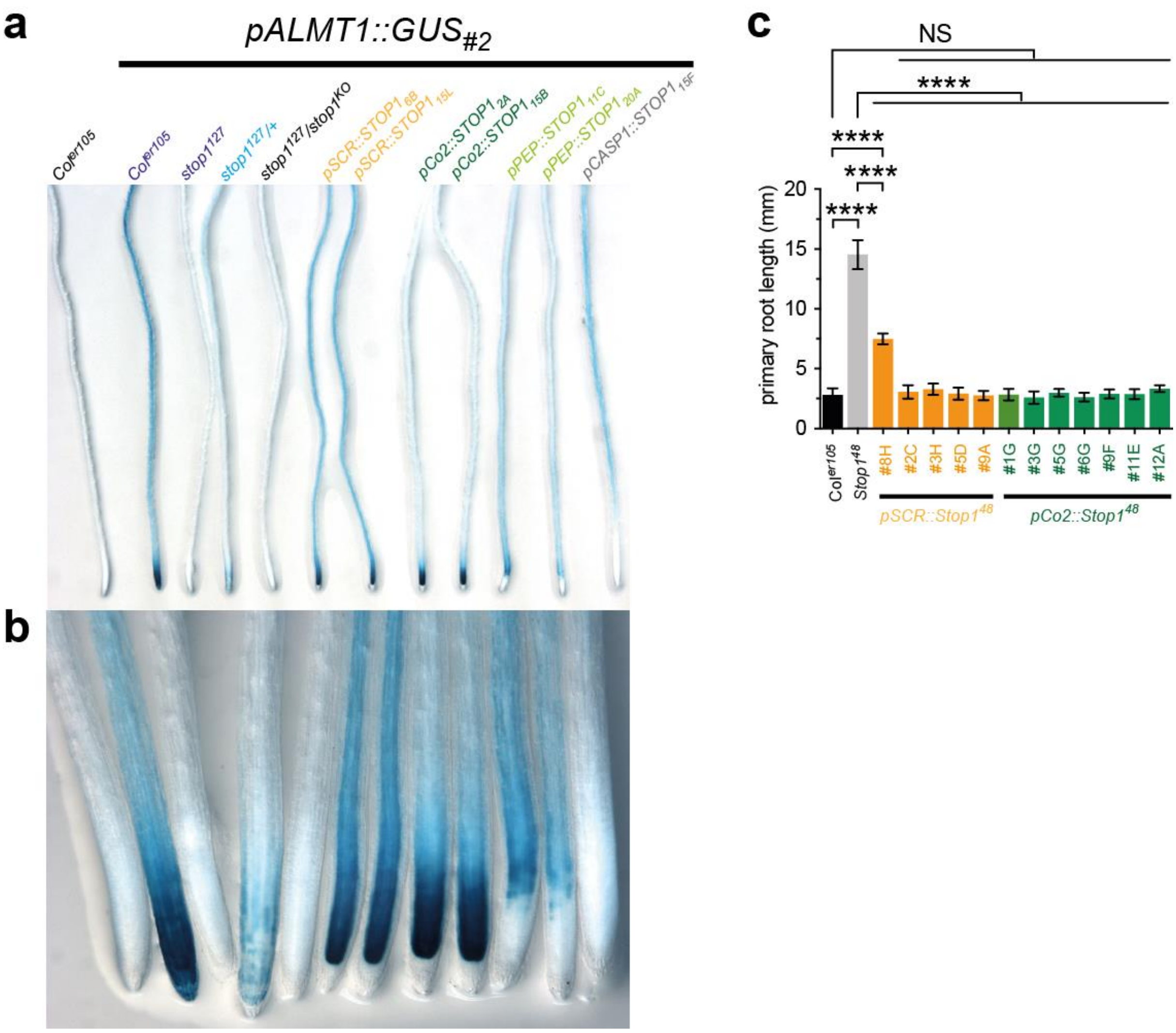


**Supplementary Figure 11: Expression of *pALMT1::GUS* in *stop1$^{KO}$* lines expressing WT *STOP1* in specific cell-types, and root length of WT lines expressing the dominant negative *Stop1$^{48}$* in the endodermis or in the cortex.**

(a,b) GUS-staining from *pALMT1::GUS*. Seedlings were grown for 5 days under +Pi prior to GUS staining (1 h). *pX::STOP1* constructs are in a *stop1$^{127}$/stop1$^{KO}$* background (F1 progeny from the cross of *stop1$^{127}$*; *pALMT1::GUS$_{\#2}$* with *stop1$^{KO}$*; *pX::STOP1*); seedlings are heterozygous for *pALMT1::GUS$_{\#2}$* except Col$^{er105}$ and *stop1$^{127}$* that are homozygous. Pictures show the primary roots (a) and magnification of the corresponding root tips (b). Three independent experiments were performed with consistent results; one representative experiment is shown.

(c) Seedlings of the indicated genotypes were grown for 6 days under –Pi and the primary root length measured. Respectively 5 and 7 independent transgenic lines for

the *pSCR::Stop1*$^{48}$ and *pCo2::Stop1*$^{48}$ constructs (in the WT Col$^{er105}$ background) are shown. The experiment has been performed three times with consistent results; one representative experiment is shown (mean +/- s.d., *n* = 12–15 seedlings per line; one-way ANOVA; **** *P*<0.0001; NS, not significant).

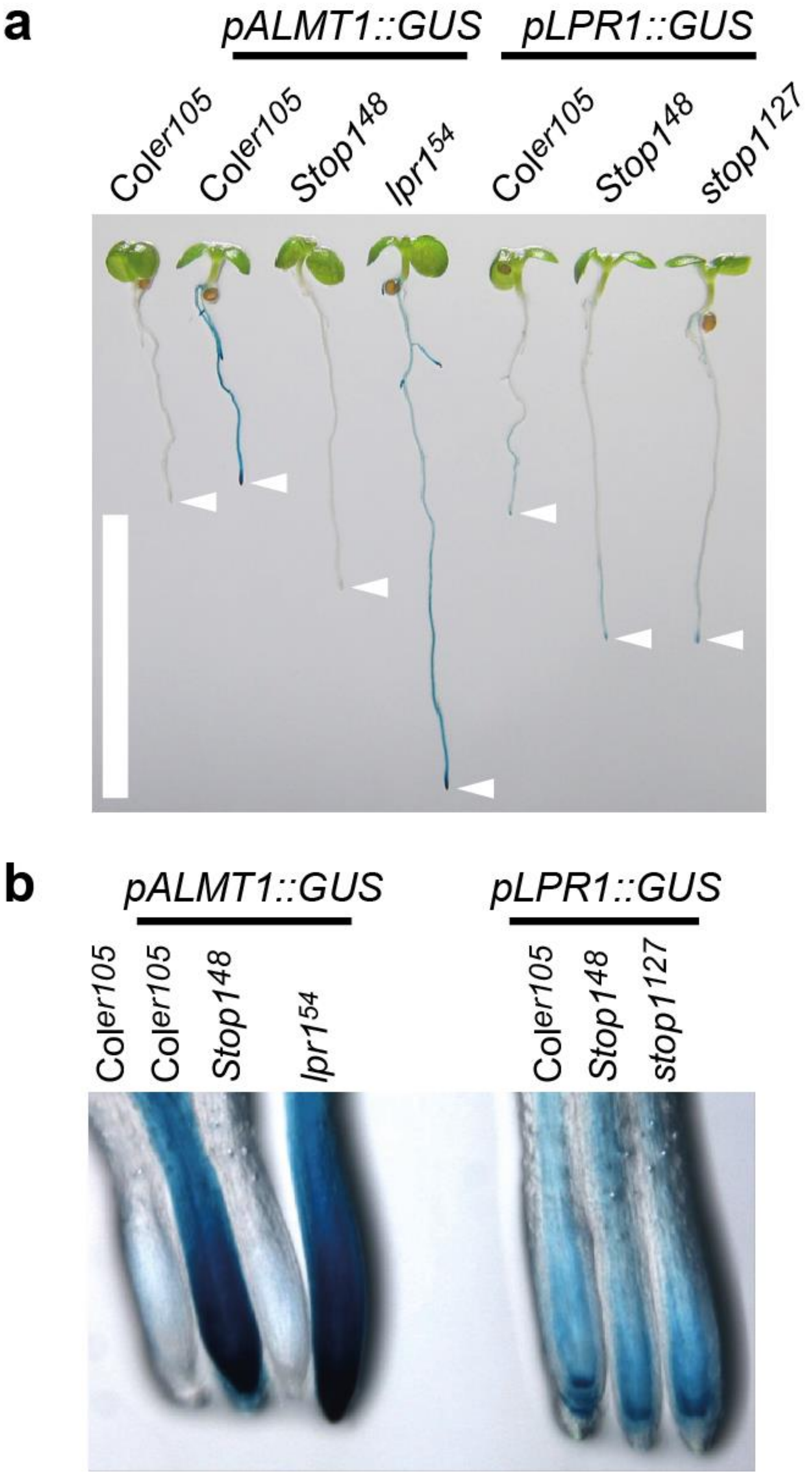


**Supplementary Figure 12: Expression of *pALMT1::GUS* in *lpr1* mutant and *pLPR1::GUS* in *stop1* mutants.**

(a) Seedlings were grown for 6 days under –Pi prior to GUS staining. The *pLPR1::GUS* (Müller et al., 2015) and *pALMT1::GUS* constructs have been

introgressed by crossing in the indicated genetics backgrounds. Arrowheads indicate the root tips. Scale bar, 1 cm.

(b) Higher magnification of the root tip.

Note that the expression of *ALMT1* is not altered in *lpr1* mutant, neither *LPR1* in *stop1* mutants. Three independent experiments were performed with consistent results; one representative experiment is shown.

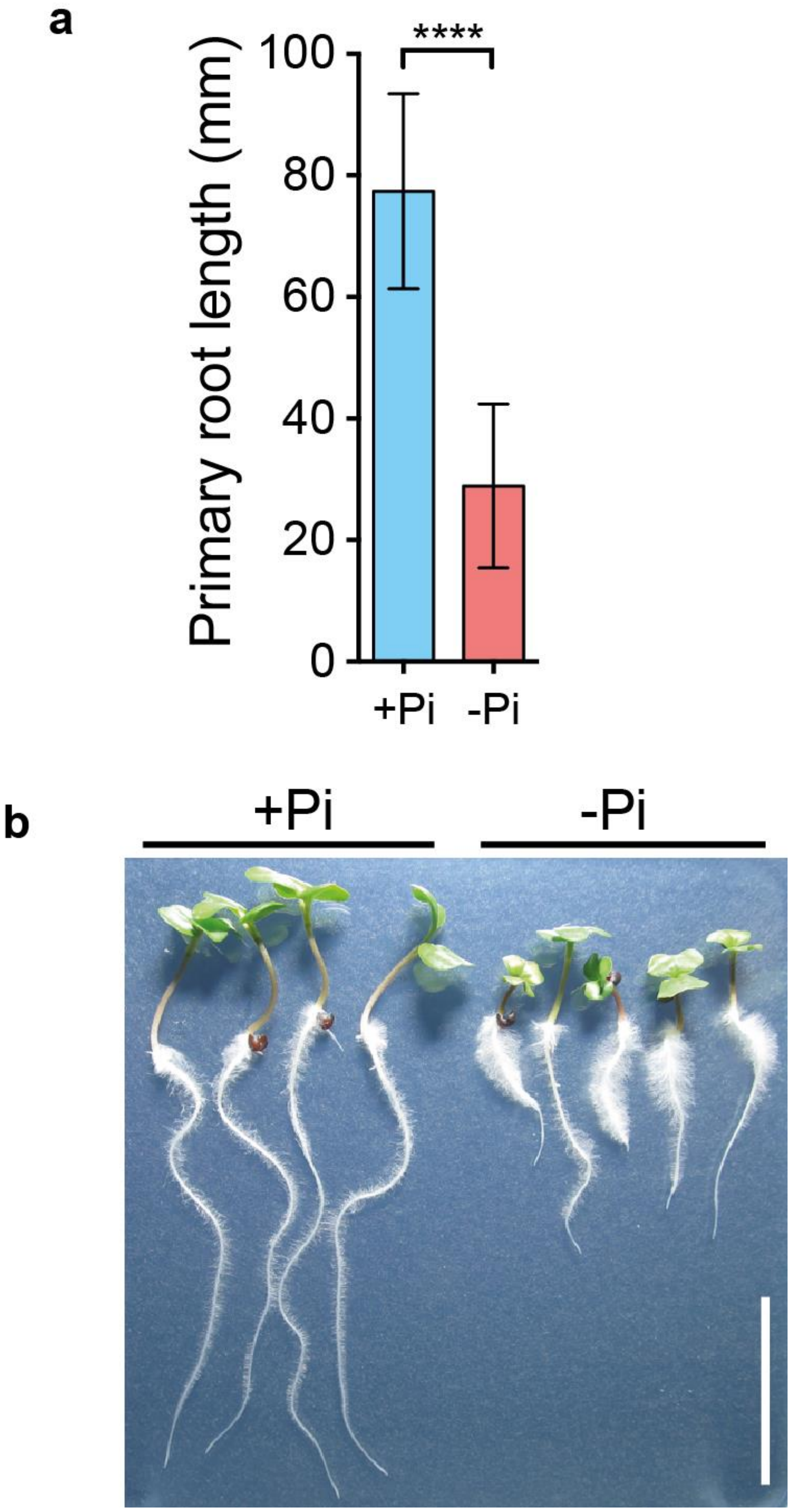
a
****
100
80
60
40
20
0
Primary root length (mm)
+Pi
-Pi
b
+Pi
-Pi

**Supplementary Figure 13: –Pi inhibits primary root growth of rapeseed seedlings.**

(a) Germinating seeds were transferred from +Pi to + or –Pi plates for 3 days before measuring the primary root length (mean +/- s.d., $n$ = 75 for +Pi and 83 for –Pi; two-tailed $t$-test; **** $P$<0.0001). Scale bar, 3 cm.

(b) Picture of representative seedlings.

Three independent experiments were performed with consistent results and one representative experiment is shown.

## Supplementary Table 1 : Primers

| name | purpose | sequence (5'>3') |
|---|---|---|
| MSAT1-2.62-F | mapping $almt1^{32}$ | CAACATCTGGACTTCCTTCCA |
| MSAT1-2.62-R | mapping $almt1^{32}$ | TGCATGTTTCATGATGACTCC |
| PMSha-2.78-F | mapping $almt1^{32}$ | ACAAATACGGCACCCATAGC |
| PMSha-2.78-R | mapping $almt1^{32}$ | TCTTCTGTCATTAGCACCCTCA |
| HRM1:1949448F | mapping $almt1^{32}$ | TGGTCTGCTGGTTGTGTACTGGCAGAGCTT |
| HRM1:1949448R | mapping $almt1^{32}$ | ACGGCTGTAGAGAGAGAGAGAGACTGGCTT |
| HRM1:2589882F | mapping $almt1^{32}$ | AGCGGCTGCTTCAGAGTAAGCACCGGG |
| HRM1:2589882R | mapping $almt1^{32}$ | AGCGCCGCCGCACGAGTTCCA |
| HRM1:3010815F | mapping $almt1^{32}$ | ACACTCTCTAGTGGCAGAGGCCTTATCCGTG |
| HRM1:3010815R | mapping $almt1^{32}$ | ACCGGAACGTGAGACCCAAGAAAGCAACT |
| HRM1:2745575L | mapping $almt1^{32}$ | CCCGGATGGCACGAACAGAGCAGCC |
| HRM1:2745575R | mapping $almt1^{32}$ | AGACCGACAGATGCGATGCGAGAAGCA |
| | | |
| MSAT1-12,22-F | mapping $almt1^{48}$ | TGTCCGGGACTGCCTTTAGC |
| MSAT1-12,22-R | mapping $almt1^{48}$ | CGCATACGTGTCACCGTGAG |
| CWI1-F | mapping $almt1^{48}$ | ACATTTTCTCAATCCTTACTC |
| CWI1-R | mapping $almt1^{48}$ | GAGAGCTTCTTTATTTGTGAT |
| | | |
| 23010cDNAF | screening *lpr1* alleles with ENDO1 | CTCTCACCCGAAGTTAATAATTCAG |
| 23010R2 | screening *lpr1* alleles with ENDO1 | CCTACGTCGGACGTTTAATC |
| 23010F4 | screening *lpr1* alleles with ENDO1 | CCCTTTCAGCTACCTACTGGC |
| RT10R | screening *lpr1* alleles with ENDO1 | CACCATCAAAACTTCGCAGAGATCGA |
| | | |
| ALMT1F1 | sequencing *ALMT1* | GAAAGTAATCAGAGAATCAG |
| ALMT1F2 | sequencing *ALMT1* | TTGGTCATGTTGGTCTTTG |
| ALMT1F3 | sequencing *ALMT1* | AGTGAATGTGAAATTGGCAG |
| ALMT1F4 | sequencing *ALMT1* | TATGTTTCTGGTTAACGTG |
| ALMT1F5 | sequencing *ALMT1* | GATCTCATTGATGACGACTG |
| ALMT1R1 | sequencing *ALMT1* | CTTGGAAACGCATGGTAATC |
| ALMT1R2 | sequencing *ALMT1* | TTCCTCTTCTCCACTACTTTG |
| ALMT1R3 | sequencing *ALMT1* | TAAATATGCAAACACCTAACA |
| ALMT1R4 | sequencing *ALMT1* | GAGAATCTATTGGCATGGC |
| ALMT1R5 | sequencing *ALMT1* | CTCTATAATCTTGCGGGTAC |
| | | |
| STOP1F1 | sequencing *STOP1* | GTATGATGAACTTTGAGCTGG |
| STOP1F2 | sequencing *STOP1* | ATTTGCCTAAGCCGGTTCTT |
| STOP1R1 | sequencing *STOP1* | ACTAGAACATCTTACATAAC |
| STOP1R2 | sequencing *STOP1* | ATGCCCTCTCATATGCATCC |
| | | |
| LBb1.3 | genotyping $stop1^{KO}$ | ATTTTGCCGATTTCGGAAC |
| pSTOP1Fw | genotyping $stop1^{KO}$ | CGGTTGAGATTAATGGG |
| pSTOP1-Fw4 | genotyping $stop1^{KO}$ | CCATTGGTGCTCTCCAAGTT |
| | | |
| SK3573_LP | genotyping $mate^{KO}$ | GACGCGGAGAGAGTTACACAG |
| SK3573_RP | genotyping $mate^{KO}$ | TCAACAATCCAACTGAGGAAAC |
| pSKI015_LP | genotyping $mate^{KO}$ | TCGTGAAGTTTCTCATCTAAGC |
| | | |
| WRKY46-F | genotyping $wrky46^{KO}$ | CCCACCAATCTCACTCAAAAGAAAA |
| WRKY46-R | genotyping $wrky46^{KO}$ | CCCTGCTGAAAAACAATGAAAAAGG |
| LBb1.3 | genotyping $wrky46^{KO}$ | ATTTTGCCGATTTCGGAAC |
| | | |
| UPB1-F | genotyping *upb1-1* | TGAATGGGAAGAAAGTTGGTG |
| UPB1-R | genotyping *upb1-1* | TTTCACAGCCCAACGTTAAAC |
| LBb1 | genotyping *upb1-1* | GCGTGGACCGCTTGCTGCAACT |
| | | |
| attB4pUBI10 | cloning *pUBQ* | GGGGACAACTTTGTATAGAAAAGTTGCCGTCGACGAGTCAGTAATAAACG |
| attB1rpUBI10 | cloning *pUBQ* | GGGGACTGCTTTTTTGTACAAACTTGCCTGTTAATCAGAAAAACTCAG |
| | | |
| attB4pALMT1 | cloning *pALMT1* | GGGGACAACTTTGTATAGAAAAGTTGCCTCCTTTTGGTTGTCTAAGCTAGACC |
| attB1rpALMT1 | cloning *pALMT1* | GGGGACTGCTTTTTTGTACAAACTTGCAACACCTTTTGATGGTCACTCAGC |
| | | |
| attB4pSTOP1 | cloning *pSTOP1* | GGGGACAACTTTGTATAGAAAAGTTGCCGGCGAAGAAGTAACTAAGGGTTGA |
| attB1rpSTOP1 | cloning *pSTOP1* | GGGGACTGCTTTTTTGTACAAACTTGCTTTTTAGTTCAAGATCTTGTTTTTCA |
| | | |
| TopoALMT1Fw | cloning *ALMT1* | CACCATGGAGAAAGTGAGAGAGATAGTG |
| TopoALMT1pasSTOPRv | cloning *ALMT1* | CTGAAGATGCCCATTACTTAATG |
| attB2rALMT1 | cloning *ALMT1* | GGGGACAGCTTTCTTGTACAAAGTGGCCATGGAGAAAGTGAGAGAGATAGTG |
| AttB3ALMT1 | cloning *ALMT1* | GGGGACAACTTTGTATAATAAAGTTGCTTACTGAAGATGCCCATTACTTAATG |

| | | |
|---|---|---|
| attB2rSTOP1 | cloning *STOP1* | GGGGACAGCTTTCTTGTACAAAGTGGCCATGGAAACTGAAGACGATTTGTG |
| AttB3STOP1 | cloning *STOP1* | GGGGACAACTTTGTATAATAAAGTTGCTTAGAGACTAGTATCTGAAACAG |
| TopoSTOP1Fw | cloning *STOP1* | CACCATGGAAACTGAAGACGATTTGTG |
| TopoSTOP1Rv | cloning *STOP1* | GAGACTAGTATCTGAAACAG |
| | | |
| Topo-GUS-Fw | cloning *GUS* | CACCATGTTACGTCCTGTAGAAACC |
| Topo-GUS-Rv | cloning *GUS* | TCATTGTTTGCCTCCCTGCTGC |
| | | |
| ROC3-F | qRT-PCR | ATCGTGATGGAGCTTTACGC |
| ROC3-R | qRT-PCR | TCGGTGAAAGCTTGATCCTT |
| STOP1-F | qRT-PCR | AAGTGGCTTTGTTCCTGTGG |
| STOP1-R | qRT-PCR | GGCTGTGTGGTTTCTTGGTT |
| ALMT1-F | qRT-PCR | GGCAGTGTGCCTACAGGATT |
| ALMT1-R | qRT-PCR | CGATTCCGAGCTCATTCTTC |
| MATE-F | qRT-PCR | GCATAGGACTTCCGTTTGTGGCA |
| MATE-R | qRT-PCR | CGAACACAAACGCTAAGGCA |
| SPX1-F | qRT-PCR | CGGGTTTTGAAGGAGATCAG |
| SPX1-R | qRT-PCR | GCGGCAATGAAAACACACTA |
| | | |
| STOP1-Fw | Yeast one-hybrid | CTTATGGGTGCTCCTCCAAAAAAGAAGAGAAAGGTAGAAACTGAAGACGATTTGTG |
| STOP1-Rv | Yeast one-hybrid | CTTATTTAATAATAAAAATCATAAATCATAAGAAATTCGCTTAGAGACTAGTATCTGAAAC |
| pALMT1-Fw | Yeast one-hybrid | ATTAGAGCTTCAATTTAATTATATCAGTTATTACCCGGGTCCTTTTGGTTGTCTAAGC |
| pALMT1-Rv | Yeast one-hybrid | CAGAAATAAGGCTAAAAAACTAATCGCATTATCATCCCTCGACAACACCTTTTGATGGTCACT |
| pALMT1-R1 | Yeast one-hybrid | CAGAAATAAGGCTAAAAAACTAATCGCATTATCATCCCTCGACCAACATACCAAAATATGATAAAC |
| pALMT1-F2 | Yeast one-hybrid | ATTAGAGCTTCAATTTAATTATATCAGTTATTACCCGGGATAGGCGGCTTCTCAGGTCG |
| pALMT1-R2 | Yeast one-hybrid | CAGAAATAAGGCTAAAAAACTAATCGCATTATCATCCCTCGACATGTTCGAATGATCTTTGCC |
| pALMT1-F3 | Yeast one-hybrid | ATTAGAGCTTCAATTTAATTATATCAGTTATTACCCGGGGGACTCAGTAAAAGAG |
| pALMTC1-Rv | Yeast one-hybrid | CAGAAATAAGGCTAAAAAACTAATCGCATTATCATCCCTCGACTTCTGTAATCGACAAGATTAG |
| pALMT1C2-Fw | Yeast one-hybrid | ATTAGAGCTTCAATTTAATTATATCAGTTATTACCCGGGCGAACATTGGAGCCCAAGTG |
| pALMT1C2-Rv | Yeast one-hybrid | CAGAAATAAGGCTAAAAAACTAATCGCATTATCATCCCTCGACCTTTAGTGAATTGTATATAGTG |
| pALMT1C3-Fw | Yeast one-hybrid | ATTAGAGCTTCAATTTAATTATATCAGTTATTACCCGGGATTCCTTAATCAAAATGTATCATGC |
| pALMT1C3-Rv | Yeast one-hybrid | CAGAAATAAGGCTAAAAAACTAATCGCATTATCATCCCTCGACTTAATTGGAGCGACGCGTGAG |
| pALMT1C4-Fw | Yeast one-hybrid | ATTAGAGCTTCAATTTAATTATATCAGTTATTACCCGGGTCTAACTAATCCAGCTCAGC |
| pALMT1C4-Rv | Yeast one-hybrid | CAGAAATAAGGCTAAAAAACTAATCGCATTATCATCCCTCGACATTTCAGGGTTAAAGTCTTAAAG |
| pALMT1C5-Fw | Yeast one-hybrid | ATTAGAGCTTCAATTTAATTATATCAGTTATTACCCGGGGGGCTAGGTTCGACTCCG |
| pALMT1C5-Rv | Yeast one-hybrid | CAGAAATAAGGCTAAAAAACTAATCGCATTATCATCCCTCGACGGATCGATGTATTTATAAAGACC |
| pALMT1C6-Fw | Yeast one-hybrid | ATTAGAGCTTCAATTTAATTATATCAGTTATTACCCGGGCATGAGTCCTAAACAAGAGTC |
